# Adenine phosphoribosyltransferase (APRT) deficiency: identification of a novel nonsense mutation

**DOI:** 10.1186/1471-2369-15-102

**Published:** 2014-07-01

**Authors:** Rea Valaperta, Vittoria Rizzo, Fortunata Lombardi, Chiara Verdelli, Marco Piccoli, Andrea Ghiroldi, Pasquale Creo, Alessio Colombo, Massimiliano Valisi, Elisabetta Margiotta, Rossella Panella, Elena Costa

**Affiliations:** 1 Research Laboratories - Molecular Biology, IRCCS Policlinico San Donato, Piazza E. Malan 2, 20097, San Donato Milanese, Milan, Italy; 2 Department Molecular Medicine, Clinical Biochemistry Unit, Foundation IRCCS Policlinico San Matteo, University of Pavia, Pavia, Italy; 3 Laboratory of Stem Cells for Tissue Engineering, IRCCS Policlinico San Donato, Milan, Italy; 4 Service Lab, Fleming Research, Milan, Italy; 5 Department of Nephrology, IRCCS Policlinico San Matteo, Pavia, Italy; 6 Service of Laboratory Medicine, IRCCS Policlinico San Donato, Milan, Italy

**Keywords:** APRT deficiency, Renal failure, Crystalline nephropathy

## Abstract

**Background:**

Adenine phosphoribosyltransferase deficiency (APRTD) is an under estimated genetic form of kidney stones and/or kidney failure, characterized by intratubular precipitation of 2,8-dihydroxyadenine crystals (2,8-DHA). Currently, five pathologic allelic variants have been identified as responsible of the complete inactivation of APRT protein.

**Case presentation:**

In this study, we report a novel nonsense mutation of the *APRT* gene from a 47- year old Italian patient. The mutation, localized in the exon 5, leads to the replacement of a cytosine with a thymine (g.2098C > T), introducing a stop codon at amino acid position 147 (p.Gln147X).

This early termination was deleterious for the enzyme structural and functional integrity, as demonstrated by the structure analysis and the activity assay of the mutant APRT protein.

**Conclusion:**

These data revealed that the p.Gln147X mutation in *APRT* gene might be a new cause of APRT disease.

## Background

Adenine phosphoribosyltransferase deficiency (APRTD) is a rare autosomal recessive metabolic disorder due to a mutation of the *APRT* gene [1]. APRT is a purine-metabolism enzyme that catalyzes the formation of 5′-adenosine monophosphate (5′-AMP) and pyrophosphate (PP) from adenine and 5-phosphoribosyl-1-pyrophosphate [2,3]. In patients with complete APRT deficiency, adenine is oxidized by xanthine oxidase (XO) to the highly insoluble and nephrotoxic derivative 2,8-dihydroxyadenine (2,8-DHA) [4], leading to urolithiasis and renal failure caused by intratubular crystalline precipitation [5,6]. The *APRT* gene, located on chromosome 16q24 [7], is approximately 2.6 kb long, contains five exons and four introns, and encodes a protein of 180 amino acid residues [8]. The human enzyme, present in a variety of cell types including erythrocyte [9], is a homodimer composed of two identical subunit species with a molecular weight of about 19.481 Da [10]. Currently, there are two isoforms produced by alternative splicing: the isoform 1 (P07741-1) and the isoform 2 (P07741-2); the isoform 1 has been considered as the ‘canonical’ one.

In the pathologic allelic variants, more than 40 mutations have been identified in the coding region of *APRT* gene in over 300 affected individuals from more than 25 countries, including at least 200 individuals from Japan. Approximately 10% of mutant alleles in affected white individuals and 5% in affected Japanese haven’t been yet identified. *APRT* gene alterations include missense, frameshift, and nonsense mutations and small deletions/insertions ranging in size from 1 to 8 base pairs. The estimated heterozygosity in different populations ranges from 0.4 to 1.2% [11], suggesting that the prevalence of a homozygous state is at least 1:50,000 to 1:100,000.

Mutant alleles responsible for the disease have been classified as APRT*Q0 for type I and APRT*J for type II APRTD. Type I APRT deficiency (complete deficiency *in vivo* or *in vitro*) has been found in patients from many different countries [12-15]. Type II deficiency (complete enzyme deficiency *in vivo* but partial deficiency in cell extracts) has been found mainly in Japan [16-18]. However, this distinction is only of historical interest, because APRT enzyme activity in intact cells has been shown to be approximately 1% in both types [19].

The most common mutations in affected European individuals are: (i) T insertion at the intron 4 splice donor site (IVS4 + 2insT) which leads to the deletion of exon 4 from the mRNA because of aberrant splicing. This mutation has been found in individuals from many European countries as well as in an affected individual from the US, (ii) A-to-T transversion in exon 3 (g.194A > T, p.Asp65Val), described in affected individuals from Iceland, Britain, and Spain. The three most common mutations in affected Japanese individuals, in order of decreasing frequency, are: (i) T-to-C missense mutation in exon 5 (g.442 T > C), (ii) G-to-A nonsense mutation in exon 3 (g.329G > A) and (iii) a four-base pair (CCGA) duplication in exon 3 that leads to a frameshift after codon 186 [20,21].

In the present study, we report the identification of a new nonsense mutation (g.2098C > T) in exon 5 (p.Gln147X) of the *APRT* gene from an Italian patient affected by APRT deficiency.

## Case presentation

### Clinical history of the patient

The patient, born in 1964, was diagnosed as affected by obstructive chronic kidney disease (CKD) with crystalluria at the age of 28. The serum creatinine was 4 mg/dl. The composition of the crystals was not investigated. Treatment with allopurinol and bicarbonate resulted in modest and transient improvement of renal function.

In 2005, the patient started hemodialysis due to end stage renal failure. In April 2010, at the age of 46, he received a kidney transplant from a deceased donor. However, the disease rapidly recurred in the transplanted organ on the 9^th^ day after the transplant and the concentrations of creatinine and urea were 7.7 mg/dl and 204 mg/dl, respectively. Two weeks after kidney transplant, a renal biopsy was performed and showed chronic tubulointerstitial nephropathy. Urinary sediment showed precipitations typical of 2,8-DHA crystals. After the diagnosis of APRT deficiency the allopurinol dose was increased to 300 mg twice a day. The patient was dismissed on May 2010 with a 2 mg/dl concentration of creatinine. In October 2010, he was again hospitalized for a bacterial lung infection. The patient’s general conditions worsened because of the onset of a multiorgan dysfunction and septic shock. The patient died in 2011, 10 months after the transplantation.

### Diagnosis of APRT deficiency

The diagnosis of APRT deficiency disease in our patient was confirmed by: (i) the absence of APRT enzyme activity in erythrocytes, (ii) the characterization of 2,8-DHA crystals in the urinary sediment and in the renal biopsy, (iii) the measurement of levels of adenine in a 24-hour urine specimen, (iv) the molecular analysis of the *APRT* gene.

High performance liquid chromatography (HPLC) analysis was used to measure APRT enzymatic activity in erythrocyte lysates and the levels of adenine were measured in 24-hour urine with UV detection [22].

The APRT activity was determined by calculating the AMP produced by hemolysates during the incubation with substrates over the basal hemolysate value, measured in a PRPP starved reaction.

**Figure 1:**
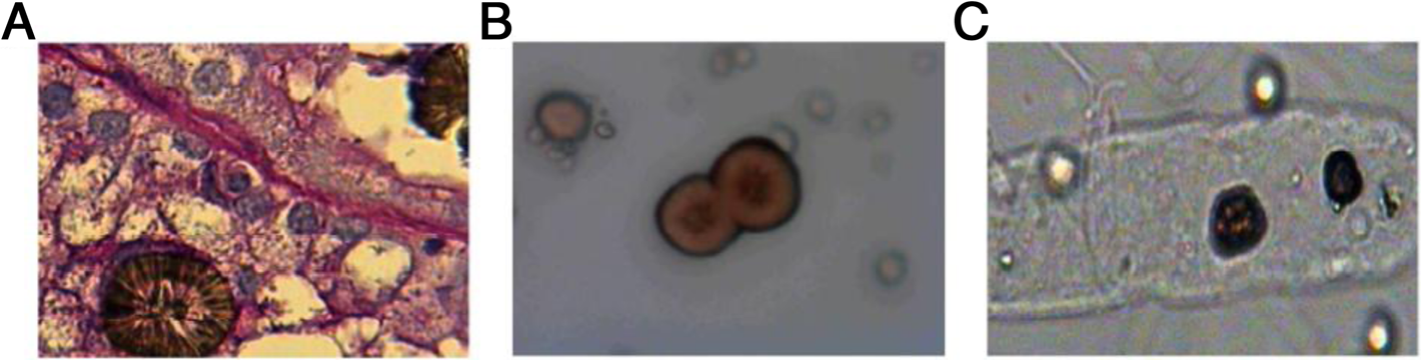
**Characterization of 2,8-DHA crystals. (A)** renal biopsy. Haematoxylin and myosin staining of a frozen section reveals interstitial inflammation surrounding multiple intra-tubular round brownish crystal that are birefringent under polarized light. **(B, C)** Urinary miscroscopy. Urinary sediment showing spherical brownish crystal that have a birefringent aqnd pseudo-Maltese cross appearance under polarized light. Magnifications: ×100 in **A**, ×40 in **B** and **C**.

The APRT activity in the patient was about 1%: residual APRT activity was 0.8 μmol/min/mg of hemoglobin while in control individuals the mean value was 36.1 ± 3.2 μmol/min/mg. The concentration of adenine in the patient’s urine was 15.68 mmol per mol of creatinine while in the control range it is 0.18-0.20 mmol per mol of creatinine.Light microscopy analysis a kidney section from a biopsy taken two weeks after transplantation, presented irregular aggregates in most of the tubules in the renal cortex. The analysis by polarized microscopy showed precipitation of 2,8-DHA crystals within tubular lumen and interstitium (Figure 1A). At the same time, all urine samples were positive for small and medium sized 2,8-DHA crystals. Microscopic examination revealed multiple spherical and reddish-brown particles with a dark outline and central spicules, that look like “Maltese crosses” under polarized light. The 2,8-DHA crystals had atypical appearances with unusual birefringence features (Figure 1B and C).

### Molecular analysis

**Figure 2:**
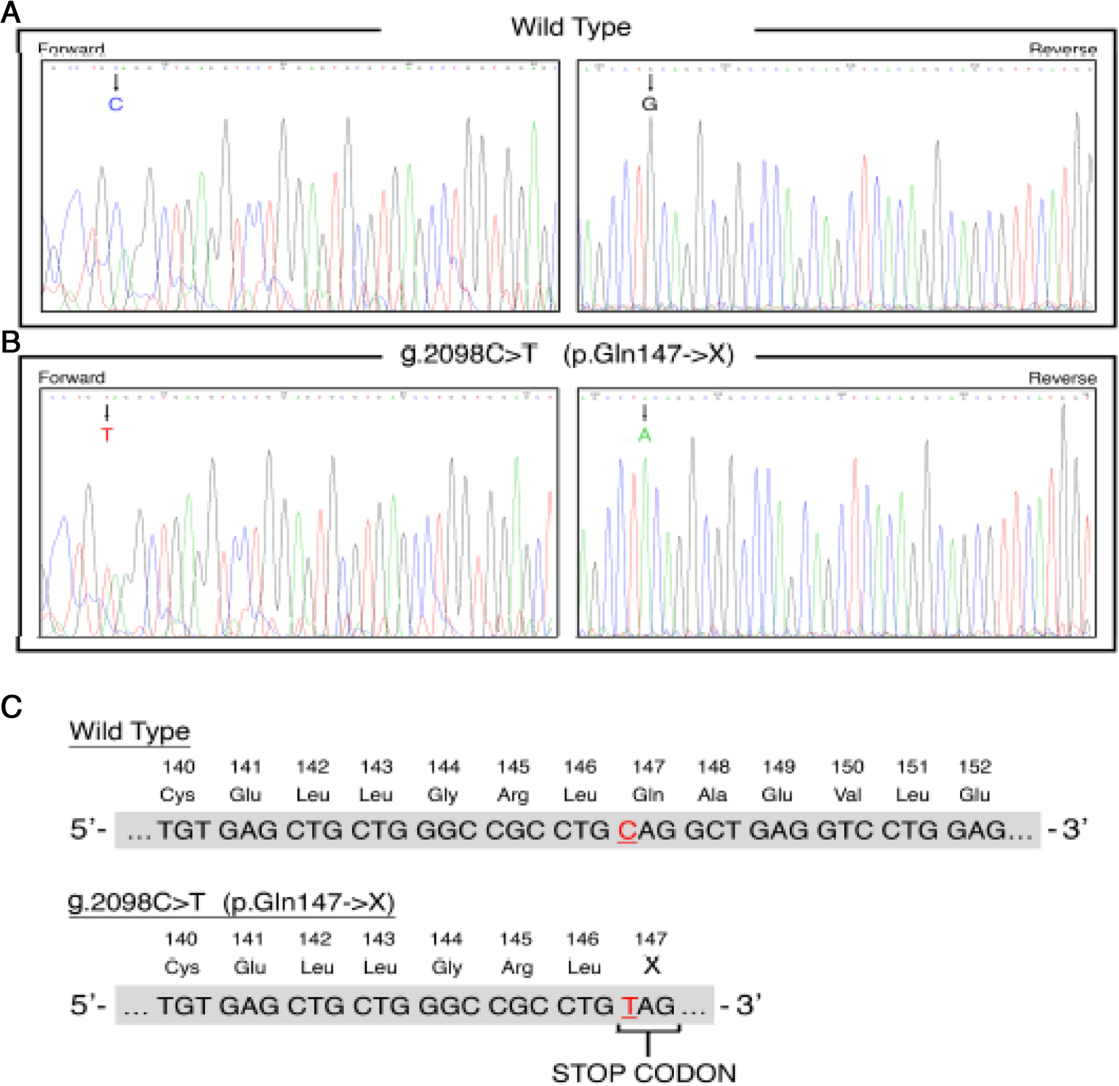
**Chromatograms of a nonsense mutation in *APRT *gene located on chromosome 16q24.** Sequence analysis of a wild type control **(A)** and of patient’s DNA **(B)**, on both strands of genomic DNA **(C)**. The sequences from the wild type control are compared with the region including the new nonsense mutation. Amino acid residues are numbered from the start codon of the open reading frame along with the nucleotide numbers. The C > T replacement is indicated in red. The position of premature termination at amino acid 147 (p.Gln147X) in the mutant patient is indicated by stop codon in the box.

Direct DNA sequencing, by Sanger method, of the five exons and their flanking regions showed that our patient was homozygous for a polymorphism in flanking region 2 named *rs8191483*. He was also carrying a homozygous nonsense mutation in exon 5 in position g.2098C > T that introduced a stop codon at amino acid position 147 (p.Gln147X). The sequence analysis of the *APRT* gene from a healthy donor was used as control (Figure 2A). The homozygous nature of this site was confirmed by the presence of only one peak in the chromatogram on both strands of genomic DNA (Figure 2B). The C > T replacement at this site leads to the formation of a truncated protein of 147 amino acids, compared to the normal 180 amino acid protein (Figure 2C). The Gln147X mutation has not been reported in previous studies, suggesting that this is a new nonsense mutation. Fifty healthy donor were used as controls. The p.Gln147X mutation was not found in any of them. The nomenclature used to describe the sequence variants was as recommended by Den Dunnen and Antonarakis [23] and by the Human Genome Variation Society (HGVS).

Unfortunately, the pedigree has not been investigated in our study since the family members refused genetic investigation.

### Protein analysis

**Figure 3:**
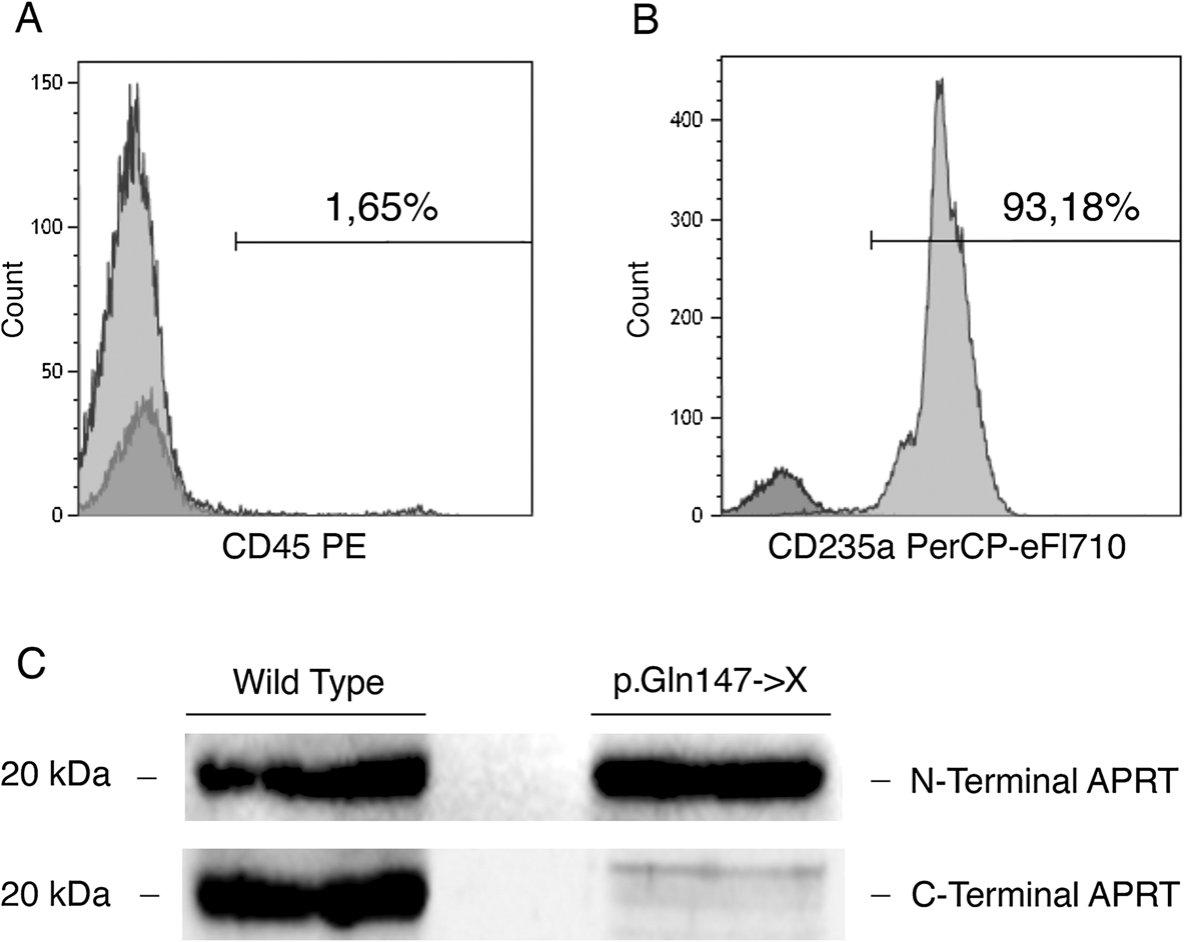
**Characterization of APRT protein in erythrocyte lysate.** Erythrocyte isolation from peripheral blood of a control and of patient by Ficoll/Paque density separation protocol. Purity was confirmed in flow cytometry, staining target cells with mouse anti-human anti-CD45 PE (leukocyte marker) **(A)** and anti-CD235a PerCP-eFluor710 (erythrocyte marker) monoclonal antibodies **(B)**. **(C)** Western blot analysis performed by C-terminal and N-terminal APRT antibodies in a wild type control and in our patient.

**Figure 4:**
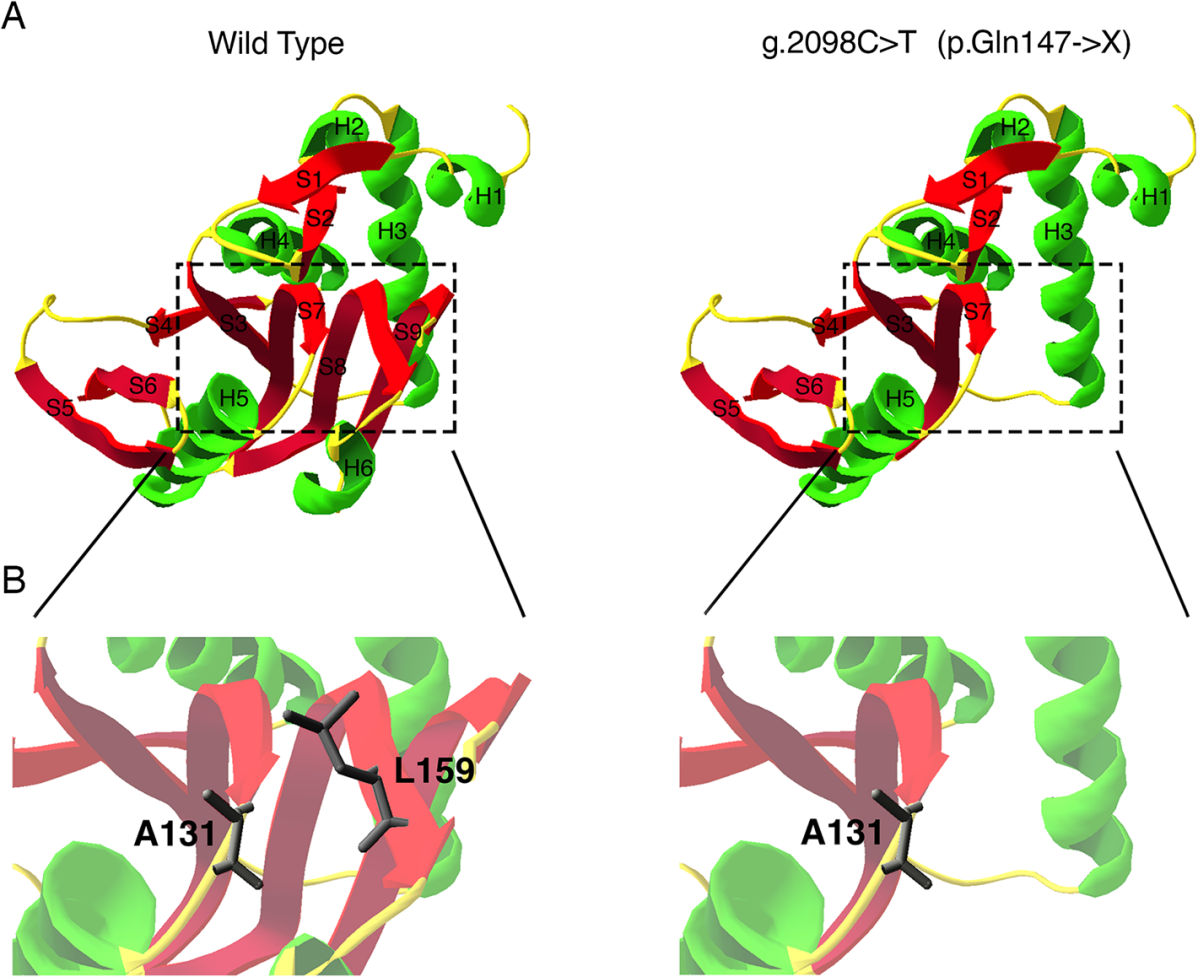
**Model of 3D structure of APRT protein. (A)** Ribbon diagram of the wild type (left) and truncated (right) APRT monomer. Truncated protein lack the C-terminal domain which includes S8 and S9 b-strands and H6 a-helix. **(B)** Calyx-like structural feature of the active site of wild type (left) and truncated (right) APRT monomer. The truncated form lack Leu159, an essential residue for the active site.

Erythrocytes isolation from peripheral blood of a control individual and from our patient was performed by Ficoll/Paque density separation protocol. Purity was confirmed by flow cytometry with a mouse anti-human-CD45 PE (Figure 3A) and anti-CD235a PerCP-eFluor710 (Figure 3B) monoclonal antibodies. SDS-PAGE protein analysis revealed the lack of the C-terminal domain in the patient sample compared to control. The N-terminal domain, used as internal control, was present in both samples (Figure 3C). Western blot analysis was performed using rabbit polyclonal APRT antibody (C-term) (Abgent, Cat AP2893b) and rabbit polyclonal APRT antibody (N-term) (Abgent, Cat AP2893a).To analyze the structural impact of the p.Gln147X nonsense mutation on APRT, the 3-D structure of the protein was modeled based on the solved protein structure (PDB ID: 1ORE) using Swiss-PdbViewer software. We reported the loss of the C-terminal of the APRT protein which includes a portion of the S8 strand, the S9 strand and the H6 α-helix, due to the new identified nonsense mutation (Figure 4A). The lack of this portion of the protein led to the removal of Leu159 residue, present in the protein active site (Figure 4B).

To evaluate the hydropathicity of both the wild type and the mutated protein, the Grand Average of Hydropathicity (GRAVY) was calculated with ProtParam Tool [24]. Loss of the C-terminal domain also caused an alteration in the protein hydrophobicity: the GRAVY of the wild type protein is 0.104, whereas the GRAVY of the mutated protein is 0.058, indicating that the mutated form of the protein is more hydrophilic than the wild type one.

## Conclusion

APRT deficiency is a disease caused by mutations in the *APRT* gene. There are more than 40 types of mutations in *APRT* gene, but currently only five allelic variants are responsible for the complete inactivation of the APRT protein both *in vitro* and *in vivo*.

This disease is characterized by the presence of 2,8-DHA crystals in urine. These crystals are radiolucent and are often considered as uric acid stones or, in renal biopsies, as oxalate crystals, causing an erroneous diagnosis. Ultraviolet or infrared spectrophotometry is required for their correct identification.

Accurate diagnosis of APRT deficiency is crucial because early treatment with allopurinol or low-purine diet effectively prevents the stone formation and may improve patients’ renal function. Nowadays, several diagnostic tools are available for the identification of the APRT deficiency such as 2,8-DHA crystal microscopic detection, APRT enzyme activity assays and molecular analysis of the *APRT* gene. In the present study, we analyzed an Italian patient with a clinical history of recurrent nephrolithiasis and chronic renal failure due to obstructive nephropathy and recurrent urinary tract infections. The patient received a renal transplant in 2010 but, unfortunately, he died 10 months after the surgical procedure. The definitive diagnosis of APRT deficiency was made by microscopic detection of 2,8-DHA crystals on the biopsy of the failing transplanted kidney and by documenting decreased APRT activity in red blood cells. Genetic analysis revealed a combination of a previously described homozygous state for *rs8191483* SNP and a novel nonsense mutation p.Gln147X. The identification of two allelic variants in homozygous state suggest the consanguinity in this family. The carriers are asymptomatic and they are usually identified during family screening.

The normal human APRT is a protein of 180 amino acids, composed of 9 β-strands and 6 α-helices, which can be divided into the “core” (residues 33–169), the “hood” (residues 5–34), and the “flexible loop” (residues 95–113) domains. On the basis of the APRT structure, which has been widely described [21], it is evident that Ala131 and Leu159 are essential residues for the specific recognition of adenine among different purines through hydrophobic interactions. Moreover, the importance of the Leu159 residue is also confirmed by the presence in the same position of a lysine residue in the PRTases that binds hypoxanthine, xanthine, or guanine. This difference between residues located at the amino acid 159 position explains the specificity of type I PRTases for their respective purines [25].

The novel nonsense mutation reported in this study caused the introduction of a stop codon leading to the loss of 33 amino acids, corresponding to the C-terminal domain of the APRT protein. This portion represents the “core” of the APRT protein. In particular, the fundamental Leu159 residue, responsible for the correct activity of the APRT enzyme, is located in the S8 strand. Therefore, the complete inactivation of the APRT protein reported in our patient is due to the impaired binding of the specific substrate to the active site, since the g.2098C > T mutation deeply affects the enzyme structural integrity.

In conclusion, the novel nonsense mutation p.Gln147X might be the main cause for the therapeutic failure observed in our patient.

Moreover, the evidence of this novel “loss-of-function” mutation might be extremely useful as a new genetic diagnostic marker for the early identification of the APRT deficiency.

More than 30 years have passed since the recognition of the first mutation in the *APRT* gene but, although much has been done during this period, the identification and characterization of new mutations might be an important step forward in the clinical practice for improving the detection of this still underdiagnosed disease.

## Consent

Written informed consent was obtained from the patient for publication of this case report and any accompanying images. A copy of the written consent is available for review by the Editor of this journal.

## Abbreviations

APRTD: Adenine phosphoribosyltransferase deficiency; APRT: Adenine phosphoribosyltransferase; 2,8-DHA: 2,8-dihydroxyadenine crystals; GRAVY: Grand average of hydropathicity; HGVS: Human Genome Variation Society.

## Competing interests

No conflicts of interest, financial or otherwise, are declared by the author(s).

## Authors’ contributions

RV, VR and FL conception and design of research; VR, FL, CV, MP, AG, PC, EM and RP performed experiments; AC and MV performed sequencing; RV, VR, FL, EC analyzed data. All authors have contributed significantly to the work and have read and approved the manuscript.

## Pre-publication history

The pre-publication history for this paper can be accessed here:

http://www.biomedcentral.com/1471-2369/15/102/prepub
